# Association between atherogenic index of plasma and periodontitis among U.S. adults

**DOI:** 10.1186/s12903-023-02853-y

**Published:** 2023-03-22

**Authors:** Jing Sun, Ge Guo

**Affiliations:** Changsha Stomatological Hospital, 389 Youyi Road, Changsha, Hunan Province 410000 China

**Keywords:** Atherogenic index of plasma, Periodontitis, NHANES database

## Abstract

**Background:**

Serum lipids have been proven to influence periodontitis. The atherogenic index of plasma (AIP) is an important marker of lipid levels. The purpose of this study was to investigate the association between periodontitis and AIP in adults.

**Methods:**

The study included participants from the 2009–2014 National Health and Nutrition Examination Survey who received a complete periodontal exam and a complete record of AIP. AIP was calculated as log_10_ (triglycerides/high-density lipoprotein cholesterol). Periodontitis can be classified into four categories based on attachment loss and probing depth (no periodontitis, moderate periodontitis, mild periodontitis, and severe periodontitis). Multivariable logistic regression after adjusting and hierarchical analysis were conducted to investigate the relationship between periodontitis and AIP in adults.

**Results:**

The final sample included 4,371 participants, representing approximately 60 million people in the United States. Periodontitis among the AIP groups (quartile, Q1-Q4) was statistically significant (*P* < 0.05). Univariate analysis showed that AIP was associated with the incidence of periodontitis (*P* < 0.05), but not with the severity of periodontitis (*P* > 0.05) in participants. Multifactorial logistic regression analysis showed no correlation between the incidence of periodontitis and AIP among all participants (the trend P-value = 0.341), but a significant association with AIP in the non-smoking participants (the trend P-value = 0.031).

**Conclusion:**

There was a significant correlation between periodontitis and AIP in the non-smoking population.

**Supplementary Information:**

The online version contains supplementary material available at 10.1186/s12903-023-02853-y.

## Introduction

Periodontitis is recognized as a chronic, multifactorial, inflammatory disease caused by the interaction of bacterial biofilms and host response [1]. The disease which includes both periodontitis and gingivitis, affects more than half of the world’s population, with more than 10% suffering from severe periodontitis [2]. It is associated with several comorbidities, such as cardiovascular disease, cancer, and respiratory disease [3]. A large-scale study recently showed that periodontitis increased the risk of coronary artery disease and all-cause mortality [4].

Both periodontitis and dyslipidemia are major risk factors for cardiovascular disease [5]. High triglycerides, low-density lipoprotein cholesterol (LDL-C), and low high-density lipoprotein cholesterol (HDL-C) are currently among the well-known lipid markers that are significantly associated with cardiovascular diseases [6]. In clinical studies, the atherogenic index of plasma (AIP), an indicator of Dyslipidemia, was defined as a more effective cardiovascular disease marker than the traditional ones [7, 8]. AIP has a significant role in a variety of diseases, including obstructive sleep apnea, bone trabecular scores in menopausal women, and even mortality in patients with new coronary artery diseases [9–11].

Serum lipids have been proven to influence the severity of periodontitis, with lipid-mediated inflammatory responses playing an important role [12, 13]. Treatment of hyperlipidemia aids in reducing the loss of periodontal disease attachment [14]. A survey of 874 Chinese older adults aged > 60 years revealed that participants with low HDL-C levels were more likely to suffer from periodontitis [15]. Similar results were detected in the Korean population, where lipid markers were found to be stronger predictors of periodontitis [16].

Therefore, in the present study, our primary objective was to elucidate the relationship between the presence and severity of periodontitis and AIP in a large nationally representative sample of adults.

## Methods

### Study population

The NHANES, National Health and Nutrition Examination Survey, is a major program of the National Center for Health Statistics that began in the early 1960s, to assess the health and nutritional status of adults and children in the United States. The survey combines household interviews and health screenings to provide comprehensive biological, psychosocial, behavioral, and demographic information free of charge. This cross-sectional analysis was conducted using data from the 2009–2014 cycle of the NHANES. The data were sourced from a database of household interviews and physical examinations. This manuscript followed Strengthening the Reporting of Observational Studies in Epidemiology (STROBE) guidelines for human observational studies. Data are de-identified, can be publicly accessed at https://www.cdc.gov/nchs/nhanes.

The study involving human participants were reviewed and approved by the NCHS. The Research Ethics Review Committee approved the NHANES survey protocol. Institutional Review Board approval and documented consent was obtained from participants (https://www.cdc.gov/nchs/nhanes/irba98.htm).

**Fig. 1:**
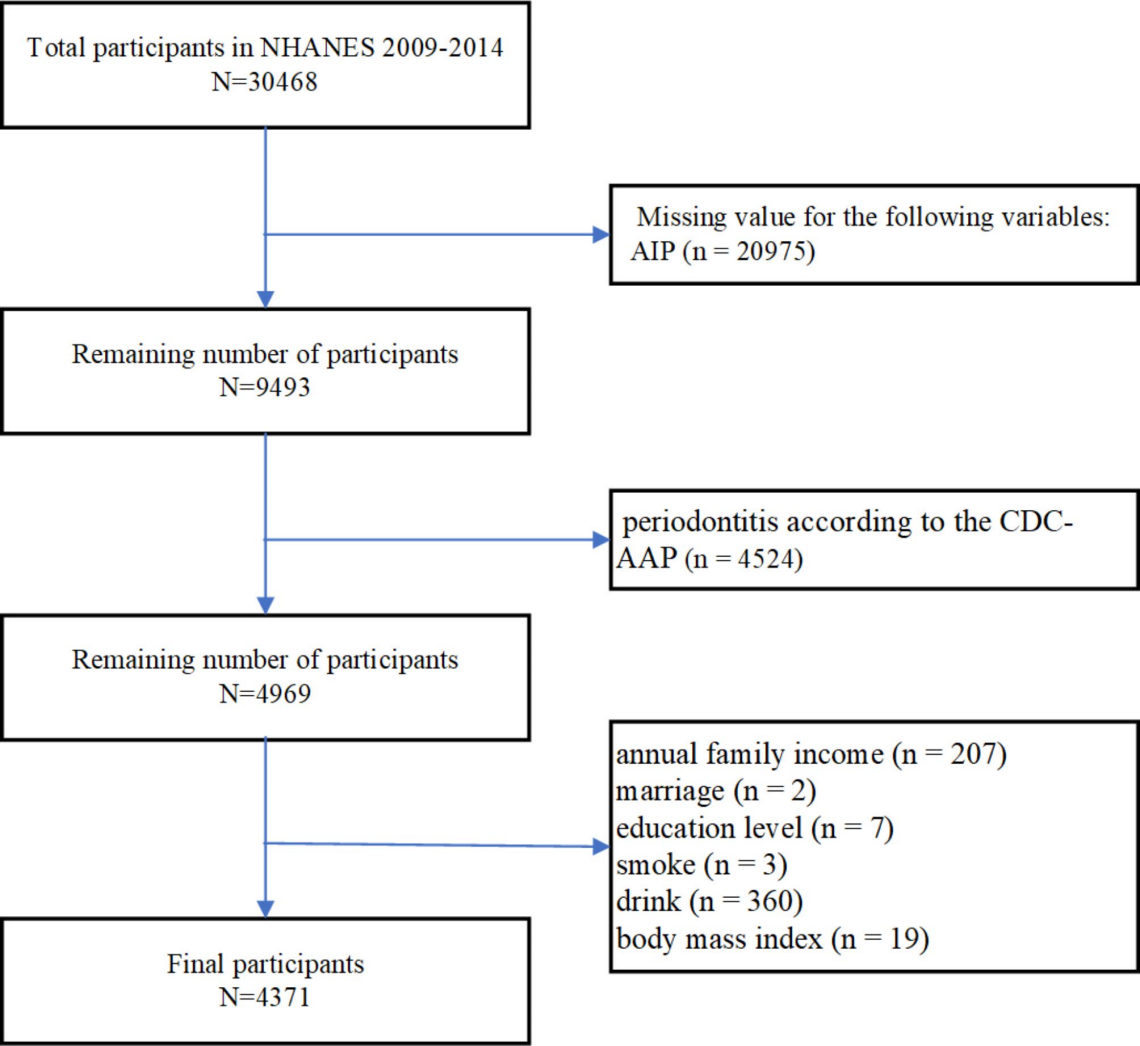
A flowchart of the participant selection process

There were 30,468 participants in the 2009–2014 cycle of NHANES, of which 26,097 were excluded from the study. Participants were excluded based on the following criteria: missing value for AIP by a blood test (n = 20,975), periodontitis according to the Centers for Disease Control and Prevention-American Academy of Periodontology (CDC-AAP) periodontitis case definitions [17] (n = 4524), annual family income (n = 207), marriage (n = 2), an education level (n = 7), smoking (n = 3), drinking (n = 360), and body mass index (n = 19). As a result, 4371 participants were enrolled in the present study for further analysis (Fig. 1). Each sample in NHANES database has its own sample weight, and “WTMEC2YR - Full sample 2 year MEC exam weight” in 2009–2010, 2011–2012, 2013–2014 cycles were selected as the designated sample weight of this study.

### Assessment of periodontitis

Participants were categorized based on the CDC-AAP periodontitis definitions [17]. Based on tooth attachment loss and probing depth, excluding third molars, the CDC-AAP has classified periodontitis into four categories: no, mild, moderate, and severe. Detailed grouping information is presented in Supplementary Table 1. In the study cohort for studying the presence of periodontitis and AIP, participants in the “no” group were categorized as “no periodontitis population”, and participants in the “mild”, “moderate”, and “severe” groups were categorized as “periodontitis populations”. In the study cohort for studying the severity of periodontitis and AIP, only patients with periodontitis were included, which compared patients with mild periodontitis to those with moderate and severe periodontitis respectively.

### Atherogenic index of plasma

AIP was assessed from a blood sample and calculated as log_10_ (triglycerides/HDL-C) and represented as a classification variable according to the quartile method.

### Covariates

The basic information of the participants was selected as the covariable for this study. Participants were divided into Non-Hispanic White, Non-Hispanic Black, Mexican American, and other race according to their ethnicity. Participants were divided into two groups, Married/Living with partner and Divorced/Never married/Widowed/Separated, based on whether they currently lived with their spouse. The Annual family income is divided into groups greater than $20,000 and groups less than or equal to $20,000. According to the level of education, participants were divided into three categories, respectively, less than high school level, high school level, and more than high school level. BMI was calculated as the weight (kg) to height squared (m^2^) ratio. Participants were categorized into three groups: “never” (lifetime use of < 100 cigarettes), “former” (lifetime use of ≥ 100 cigarettes and no longer smoking), or “now” (lifetime use of ≥ 100 cigarettes and smoking every day or someday)[18]. Participants were divided into five groups based on drinking history: never, former, mild, moderate, and heavy [19].

### Statistical analysis

The primary objective was to investigate whether the presence and severity of periodontitis were associated with AIP in a large-scale population survey in the United States. All participants were divided into four groups according to the previous classification method of periodontitis: no, moderate, mild, and severe. AIP (continuous variables) was represented by quartiles in the study. Complex data were calculated using descriptive statistics. Categorical variables among covariates were calculated as unweighted frequencies and weighted estimates of overall proportions, and continuous variables were presented as mean ± standard error. Baseline data for study participants were grouped using the AIP quartile. The chi-square test or Student’s t-test was used to determine the P-value of the distribution. Univariate logistic regression was performed to evaluate the correlation between the presence and severity of periodontitis and AIP with other covariates. Furthermore, stratified analysis was used to assess the relationship between the presence and severity of periodontitis and AIP according to age, sex, BMI, smoking, and alcohol consumption. Multinomial regression analyses were conducted to investigate the correlation between the presence of periodontitis and AIP. Models were adjusted for age, sex, BMI, ethnicity, annual family income, education, smoking, and drinking. All statistical analyses in this study were carried out using the R version 4.1.2 (NHANESR package). *P* = 0.05 was used to determine the statistical significance.

## Results

The weighted characteristics of the analyzed samples are shown in Table 1. The final sample included 4371 participants, 2191 (50.1%) men, and 2180 (49.9%) women, aged 30–80 years, representing 60 million people in the United States. AIP ranged from − 1.09 to 1.70, with a median of -0.05. Patients with periodontitis accounted for 50% of the total population, among which 4% had mild, 35% had moderate, and 11% had severe periodontitis. Statistically significant differences (*P* ≤ 0.05) were detected between periodontitis and AIP. Covariates with significant differences included age, sex, BMI, race, marriage, education, smoking, and alcohol consumption.

**Table 1:** Baseline characteristics of participants with different AIPs

	Atherogenic Index of Plasma				
Variable	Q1 (-1.093, -0.266)	Q2 (-0.266, -0.049)	Q3 (-0.049, 0.178)	Q4 (0.178, 1.698)	*P*
	N = 1093 (25.01%)	N = 1092 (24.98%)	N = 1095 (25.05%)	N = 1091 (24.96%)	
Age	51.38(0.63)	51.16(0.49)	52.02(0.43)	50.31(0.47)	0.05
Sex					< 0.0001
Male	372(32.02)	535(48.15)	573(54.52)	711(66.58)	
Female	721(67.98)	557(51.85)	522(45.48)	380(33.42)	
BMI					< 0.0001
<=25	498(48.25)	314(28.11)	215(19.33)	136(10.44)	
25–30	338(32.05)	402(39.44)	369(35.01)	402(34.71)	
>30	257(19.70)	376(32.46)	511(45.66)	553(54.85)	
Ethnicity					< 0.0001
Non-Hispanic White	491(71.17)	507(72.30)	471(68.39)	545(73.77)	
Non-Hispanic Black	318(14.64)	230(10.39)	192(8.58)	107(4.87)	
Mexican American	89(4.89)	135(6.78)	191(9.53)	200(9.56)	
Other Race	195(9.31)	220(10.53)	241(13.49)	239(11.80)	
Marriage					0.86
Married/Living with partner	686(70.78)	709(70.25)	740(70.57)	732(68.73)	
Divorced/Never married/Widowed/Separated	407(29.22)	383(29.75)	355(29.43)	359(31.27)	
Annual family income					0.002
<=20,000	450(44.19)	418(38.06)	400(34.11)	420(34.39)	
>20,000	643(55.81)	674(61.94)	695(65.89)	671(65.61)	
Education					< 0.0001
Less than high school	178(10.16)	235(13.99)	263(16.92)	311(18.92)	
High school	212(16.32)	233(20.28)	249(22.74)	246(22.74)	
More than high school	703(73.52)	624(65.73)	583(60.34)	534(58.34)	
Smoke					< 0.0001
Never	673(63.35)	648(60.62)	601(52.80)	538(50.64)	
Former	266(25.44)	260(25.07)	300(29.44)	286(26.04)	
Now	154(11.21)	184(14.31)	194(17.76)	267(23.32)	
Drink					< 0.0001
Never	147(10.35)	134(9.49)	181(13.07)	130(8.20)	
Former	151(11.01)	180(14.26)	209(15.56)	224(17.59)	
Mild	418(41.83)	454(43.82)	361(36.59)	344(34.50)	
Moderate	208(22.16)	146(15.49)	145(15.97)	136(16.61)	
Heavy	169(14.65)	178(16.94)	199(18.80)	257(23.10)	
Periodontitis					0.002
No	627(66.40)	549(59.19)	526(55.29)	499(54.58)	
Mild	39(3.12)	44(3.91)	54(4.44)	54(4.75)	
Moderate	333(25.60)	390(29.85)	390(31.67)	403(31.33)	
Severe	94(4.88)	109(7.05)	125(8.60)	135(9.34)	

Univariate logistic regression analysis showed that AIP was associated with the incidence of periodontitis (Table 2). The odds ratio (OR) values and 95% confidence intervals for the risk of periodontitis in groups Q2, Q3, and Q4 in comparison to group Q1 were 1.36 (1.07, 1.74), 1.60 (1.24, 2.06), and 1.64 (1.26, 2.14), respectively. AIP was not associated with the severity of periodontitis (*P* > 0.05) but was positively associated with the risk of periodontitis. In addition, the severity of periodontitis was found to be significantly associated with smoking, alcohol consumption, and education level (Table 2).

**Table 2:** Univariate analysis of variables associated with the presence and severity of periodontitis

	Presence		Severity	
Variable	95% CI	*P*	95% CI	P
Age	1.04(1.03,1.05)	< 0.0001	1.04(1.03,1.06)	< 0.0001
Sex				
Male	ref	ref	ref	ref
Female	0.55(0.48,0.63)	< 0.0001	0.77(0.50,1.18)	0.22
BMI				
<=25	ref	ref	ref	ref
25–30	1.01(0.82,1.25)	0.93	0.64(0.40,1.03)	0.06
>30	1.16(0.95,1.42)	0.15	0.51(0.29,0.91)	0.02
Ethnicity				
Non-Hispanic White	ref	ref	ref	ref
Non-Hispanic Black	2.17(1.64,2.87)	< 0.0001	1.03(0.66,1.60)	0.89
Mexican American	2.49(1.84,3.36)	< 0.0001	0.88(0.48,1.59)	0.66
Other Race	1.42(1.08,1.85)	0.01	1.66(0.86,3.19)	0.12
Marriage				
Married/Living with partner	ref	ref	ref	ref
Divorced/Never married/Widowed/Separated	1.58(1.36,1.84)	< 0.0001	0.95(0.69,1.30)	0.73
Annual family income				
<=20,000	ref	ref	ref	ref
>20,000	1.24(1.05,1.46)	0.01	0.97(0.59,1.59)	0.89
Education				
Less than high school	ref	ref	ref	ref
High school	0.55(0.44,0.68)	< 0.0001	0.58(0.34,1.01)	0.05
More than high school	0.25(0.20,0.31)	< 0.0001	0.42(0.27,0.66)	< 0.001
Smoke				
Never	ref	ref	ref	ref
Former	1.67(1.33,2.10)	< 0.0001	2.65(1.63,4.30)	< 0.001
Now	2.98(2.39,3.72)	< 0.0001	2.79(1.70,4.57)	< 0.001
Drink				
Never	ref	ref	ref	ref
Former	1.30(0.96,1.77)	0.09	0.68(0.35,1.33)	0.25
Mild	0.72(0.52,1.00)	0.05	0.52(0.30,0.91)	0.02
Moderate	0.57(0.39,0.82)	0.004	0.34(0.16,0.75)	0.01
Heavy	1.10(0.72,1.68)	0.65	0.47(0.26,0.86)	0.01
AIP				
Q1	ref	ref	ref	ref
Q2	1.36(1.07,1.74)	0.01	0.97(0.51,1.83)	0.91
Q3	1.60(1.24,2.06)	< 0.001	0.93(0.54,1.61)	0.79
Q4	1.64(1.26,2.14)	< 0.001	0.88(0.50,1.54)	0.64

The relationship between periodontitis and AIP was examined using multifactorial analysis (Supplementary Table 2). Groups with low AIP values (Q1) were less likely to develop periodontitis than Q2, Q3, and Q4 (the trend P-value < 0.001) in the unadjusted model (model 1). While age and gender were adjusted in model 2. The trend P-value in both models was consistent with that in the unadjusted model group, indicating that participants with low AIP values were less likely to develop periodontitis. Notably, after adjusting all covariates in Table 1 in model 3, the trend P-value was 0.341, and the P-values of all groups were > 0.05 as compared to group Q1. This indicated that there was no significant correlation between periodontitis and AIP in all participants.

Further, stratified analyses were performed based on age, sex, BMI, smoking, and drinking (Table 3). In the age-stratified analysis, only the 30–40 years and 40–50 years age groups with periodontitis were positively associated with AIP (the trend P-value < 0.05). In the sex-stratified analysis, the incidence of periodontitis was associated with AIP only in female participants, and the OR values and 95% confidence intervals of groups Q2, Q3, and Q4 were 1.441 (1.000, 2.078), 1.674 (1.188, 2.359) and 1.650 (1.168, 2.331) respectively, compared to group 1. In the stratified analysis between BMI groups, significant differences in the prevalence of periodontitis in different groups of AIP were detected in BMI ( < = 25) and BMI (25–30) (the trend P-value = 0.01). In the lifestyle habits-stratified analysis, we found that periodontitis was significantly associated with AIP in never-smoking participants (the trend P-value < 0.001).

**Table 3:** Stratified analyses of the relationship between AIP and periodontitis according to age, sex, BMI, smoking, and alcohol consumption

	Atherogenic Index of Plasma							
	Q1	Q2	*P*	Q3	*P*	Q4	*P*	*P* for trend
Age								
30–40	Reference	1.187(0.775,1.818)	0.422	1.553(1.017,2.371)	0.042	1.751(1.183,2.592)	0.006	0.003
40–50	Reference	2.149(1.529,3.021)	< 0.0001	2.834(1.709,4.700)	< 0.001	3.190(2.005,5.075)	< 0.0001	< 0.0001
50–60	Reference	1.179(0.736,1.890)	0.485	1.400(0.773,2.537)	0.260	1.401(0.833,2.357)	0.198	0.188
60–70	Reference	1.782(0.890,3.569)	0.101	1.545(0.837,2.853)	0.160	1.732(0.786,3.817)	0.169	0.199
70–80	Reference	0.892(0.540,1.473)	0.647	0.816(0.486,1.372)	0.435	0.627(0.344,1.141)	0.123	0.09
Sex								
Male	Reference	1.002(0.700,1.435)	0.990	1.125(0.765,1.654)	0.542	1.091(0.767,1.553)	0.621	0.51
Female	Reference	1.441(1.000,2.078)	0.050	1.674(1.188,2.359)	0.004	1.650(1.168,2.331)	0.005	0.003
BMI								
<=25	Reference	1.308(0.909,1.884)	0.145	1.900(1.161,3.110)	0.012	1.680(0.874,3.227)	0.117	0.01
25–30	Reference	1.459(0.940,2.264)	0.090	1.438(0.837,2.471)	0.183	1.885(1.214,2.927)	0.006	0.01
> 30	Reference	1.297(0.813,2.070)	0.268	1.555(0.995,2.431)	0.053	1.444(0.904,2.306)	0.121	0.095
smoke								
never	Reference	1.440(1.066,1.945)	0.018	1.788(1.290,2.479)	< 0.001	1.920(1.361,2.709)	< 0.001	< 0.001
former	Reference	1.199(0.791,1.819)	0.385	1.155(0.757,1.764)	0.496	1.205(0.815,1.782)	0.342	0.435
now	Reference	1.175(0.593,2.325)	0.637	1.146(0.675,1.945)	0.607	0.874(0.446,1.710)	0.687	0.527
drink								
never	Reference	1.474(0.750,2.898)	0.253	1.769(0.941,3.326)	0.075	1.806(0.995,3.280)	0.052	0.044
former	Reference	1.065(0.648,1.752)	0.799	1.877(1.041,3.387)	0.037	1.848(1.040,3.284)	0.037	0.018
mild	Reference	1.314(0.869,1.989)	0.191	1.200(0.771,1.870)	0.411	1.320(0.863,2.017)	0.195	0.268
moderate	Reference	1.934(1.154,3.241)	0.014	1.941(1.100,3.427)	0.023	2.296(1.335,3.948)	0.003	0.005
heavy	Reference	1.000(0.627,1.593)	0.999	1.390(0.787,2.458)	0.250	1.137(0.670,1.929)	0.628	0.462

The most significant differences were found in the 40–50 years age group (the trend P-value < 0.001) in the age-stratified analysis, and the non-smoking patients (the trend P-value < 0.001) in the smoking-stratified analysis. Subsequently, we analyzed the relationship between periodontitis and AIP using multifactorial logistic regression in the non-smoking (Table 4) and 40–50 years age groups (Supplementary Table 3). After adjusting all covariates, the trend p-value for the relationship between periodontitis and AIP was 0.031 and 0.162 in the non-smoking participants and 40–50 age group participants respectively. This showed that the prevalence of periodontitis was significantly associated with AIP in the non-smoking population.

**Table 4:** Adjusted multinomial logistic regression of AIP with periodontitis in a non-smoking population

	Q1	Q2		Q3		Q4		*p* for trend
		OR (95% CI)	*P*	OR (95% CI)	*P*	OR (95% CI)	*P*	
The presence of periodontitis								
Model 1	Reference	1.440(1.066,1.945)	0.018	1.788(1.290,2.479)	< 0.001	1.920(1.361,2.709)	< 0.001	< 0.001
Model 2	Reference	1.374(0.994,1.899)	0.054	1.600(1.107,2.315)	0.014	1.769(1.216,2.574)	0.004	0.003
Model 3	Reference	1.310(0.890,1.929)	0.165	1.347(0.881,2.060)	0.162	1.672(1.066,2.624)	0.027	0.031

Model 1: Unadjusted

Model 2: Adjusted for age and sex

Model 3: Adjusted for age, sex, BMI, ethnicity, annual family income, education, and drinking

## Discussion

Periodontitis is the most common oral disease and the sixth most prevalent human disease [20]. Therefore, research into how periodontitis affects bodily functions is crucial for the public health domain. A popular topic among these is the relationship between periodontitis and blood lipid. Studies have shown that patients with periodontitis have significantly lower HDL-C [21, 22]. A meta-analysis also showed that participants with periodontitis had lower HDL-C levels than those without the condition [22]. Other studies have, however, found no significant correlation between periodontitis and HDL-C [23, 24]. The heterogeneity of these results could be attributed to differences in sample size and participant characteristics and warrants a larger sample size of survey results for verification.

AIP has been used to quantify comprehensive lipid levels as a reliable biomarker of dyslipidemia [7]. Studies have shown that AIP is associated with a variety of biological events, including obstructive sleep apnea, bone trabecular scores in menopausal women, and even mortality in patients with new coronary arteries [9–11]. It is also considered a biomarker for coronary syndrome and metabolic syndrome and is positively associated with the risk of cardiovascular diseases [25].

Based on the above information, we concluded that there may be a possible link between periodontitis and AIP. The traditional single-center, small-sample population approach to evaluate the relationship between periodontitis and lipid levels is still controversial and has low predictive value. In contrast, our study could obtain relatively reliable results using data from a national population survey.

In our study, AIP was used as four categorical variables. The results of the univariate logistic analysis showed a significant association between the presence of periodontitis and AIP, but not between the severity of periodontitis and AIP. The results of our multifactorial logistic analysis, controlling all covariates, showed no significant relationship between the presence of periodontitis and AIP. To investigate whether this correlation exists in a specific population, we used the stratified analyses and found significant differences in periodontal disease prevalence between different AIP groups among the non-smoking patient group and the 40–50 age group, respectively. After controlling all covariates, multivariate logistic regression showed a significant association between the presence of periodontitis and AIP in the non-smoking population.

Our results are similar to the previously reported association between periodontitis and dyslipidemia. A meta-analysis also showed that periodontal disease was associated with hyperlipidemia [26]. Gomes-Filho et al. showed that patients with periodontitis were 1.47 times more likely to have a TG/HDL-C ratio ≥ 2.3 than patients without periodontitis, indicating a positive association between periodontitis and dyslipidemia [27]. However, our results showed that this correlation was only present in non-smoking participants. This may be due to the fact that smoking can affect both serum lipids and periodontitis [28, 29]. In non-smoking patients, the correlation between periodontitis and AIP better reflects their close link.

We speculate that in patients with periodontitis, the release of inflammatory mediators from periodontal tissues into the bloodstream may cause systemic low-intensity inflammation, which can adversely affect health [30]. This systemic inflammation interferes with HDL-C production while increasing LDL-C and triglyceride levels [31]. Moreover, changes in lipoproteins and lipids may affect the development of several systemic disease processes, while the inflammatory response of the body caused due to periodontitis may alter the body’s immune response through multiple mechanisms [32, 33].

As a result, the findings of the present study provide a theoretical basis for offering information on periodontitis and body lipid levels. The main advantage of this study is that we were able to obtain relatively reliable data and reduce bias by analyzing a large sample of the population. Another advantage of this study is that we obtained more reliable results by determining the relationship between discriminating AIP and periodontitis in different populations through stratified analyses.

Despite the study’s novel findings from a large US population sample, it has several limitations. First, because the study used a cross-sectional design, we were unable to determine the complex causal relationship between periodontitis and AIP. Second, it was unable to exclude all confounding factors that could impact the results, such as diabetes, hypertension, arrhythmia, fatty liver, and other diseases that can affect lipid abnormalities. Because retrospective studies are very difficult to accurately diagnose related diseases, this study did not include relevant contents. Third, the reason for the significant correlation between periodontitis and AIP in the non-smoking population needs to be investigated further.

## Conclusion

The present study found a significant correlation between periodontitis and AIP in the non-smoking population.

## Electronic supplementary material

Below is the link to the electronic supplementary material.

Supplementary Table 1. Periodontitis is classified according to the US CDC-AAP

Supplementary Table 2. Adjusted multinomial logistic regression of AIP with periodontitis

Supplementary Table 3. Adjusted multinomial logistic regression of AIP with periodontitis in a population aged 40-50 years

## Data Availability

The data are available from the website (https://www.cdc.gov/nchs/nhanes/index.htm).

## Abbreviations

AIP: Atherogenic index of plasma
HDL-C: high-density lipoprotein cholesterol
LDL-C: low-density lipoprotein cholesterol
BMI: body mass index
NHANES: National Health and Nutrition Examination Survey
CDC-AAP: Centers for Disease Control and Prevention- American Academy of Periodontology

## References

[1] Kornman KS (2008). Mapping the pathogenesis of periodontitis: a new look. J Periodontol.

[2] Janakiram C, Dye BA (2020). A public health approach for prevention of periodontal disease. Periodontol 2000.

[3] Slots J. Periodontitis: facts, fallacies and the future.Periodontology 20002017, 75(1):7–23. 10.1111/prd.12221 28758294

[4] Stewart R, West M (2016). Increasing evidence for an Association between Periodontitis and Cardiovascular Disease. Circulation.

[5] Bora K, Pathak MS, Borah P, Hussain MI, Das D (2017). Association of the apolipoprotein A-I gene polymorphisms with Cardiovascular Disease Risk factors and atherogenic indices in patients from Assam, Northeast India. Balkan J Med genetics: BJMG.

[6] Manninen V, Tenkanen L, Koskinen P, Huttunen JK, Mänttäri M, Heinonen OP, Frick MH (1992). Joint effects of serum triglyceride and LDL cholesterol and HDL cholesterol concentrations on coronary heart disease risk in the Helsinki Heart Study. Implications for treatment. Circulation.

[7] Fernández-Macías JC, Ochoa-Martínez AC, Varela-Silva JA, Pérez-Maldonado IN (2019). Atherogenic index of plasma: Novel Predictive Biomarker for Cardiovascular Illnesses. Arch Med Res.

[8] Wu J, Zhou Q, Wei Z, Wei J, Cui M (2021). Atherogenic index of plasma and coronary artery disease in the Adult Population: a Meta-analysis. Front Cardiovasc Med.

[9] Bikov A, Meszaros M, Kunos L, Negru AG, Frent SM, Mihaicuta S. Atherogenic Index of Plasma in Obstructive Sleep Apnoea.Journal of clinical medicine2021, 10(3). 10.3390/jcm10030417 PMC7865393 33499142

[10] Hernández JL, Olmos JM, Pariente E, Ramos C, Martínez J, Nan D (2021). The atherogenic index of plasma is related to a degraded bone microarchitecture assessed by the trabecular bone score in postmenopausal women: the Camargo Cohort Study. Maturitas.

[11] Turgay Yıldırım Ö, Kaya Ş (2021). The atherogenic index of plasma as a predictor of mortality in patients with COVID-19. Heart & lung: the journal of critical care.

[12] Nibali L, Rizzo M, Li Volti G, D’Aiuto F, Giglio RV, Barbagallo I, Pelekos G, Donos N (2015). Lipid subclasses profiles and oxidative stress in aggressive periodontitis before and after treatment. J Periodontal Res.

[13] Lee CT, Li R, Zhu L, Tribble GD, Zheng WJ, Ferguson B, Maddipati KR, Angelov N, Van Dyke TE (2021). Subgingival Microbiome and Specialized pro-resolving lipid mediator pathway profiles are correlated in Periodontal inflammation. Front Immunol.

[14] Magán-Fernández A, Papay-Ramírez L, Tomás J, Marfil-Álvarez R, Rizzo M, Bravo M, Mesa F (2014). Association of simvastatin and hyperlipidemia with periodontal status and bone metabolism markers. J Periodontol.

[15] Zhu H, Ye G, Xie Y, Zhu K, Zhu F, Chen Q (2022). Association of high-density lipoprotein cholesterol and periodontitis severity in chinese elderly: a cross-sectional study. Clin Oral Invest.

[16] Lee YC, Lee JW, Kwon YJ (2022). Comparison of the triglyceride glucose (TyG) index, triglyceride to high-density lipoprotein cholesterol (TG/HDL-C) ratio, and metabolic score for insulin resistance (METS-IR) associated with periodontitis in korean adults. Therapeutic Adv chronic disease.

[17] Eke PI, Page RC, Wei L, Thornton-Evans G, Genco RJ (2012). Update of the case definitions for population-based surveillance of periodontitis. J Periodontol.

[18] Gay IC, Tran DT, Paquette DW (2018). Alcohol intake and periodontitis in adults aged ≥ 30 years: NHANES 2009–2012. J Periodontol.

[19] Rattan P, Penrice DD, Ahn JC, Ferrer A, Patnaik M, Shah VH, Kamath PS, Mangaonkar AA, Simonetto DA (2022). Inverse Association of Telomere length with Liver Disease and Mortality in the US Population. Hepatol Commun.

[20] Kassebaum NJ, Bernabé E, Dahiya M, Bhandari B, Murray CJ, Marcenes W (2014). Global burden of severe periodontitis in 1990–2010: a systematic review and meta-regression. J Dent Res.

[21] Liu J, Wu Y, Ding Y, Meng S, Ge S, Deng H (2010). Evaluation of serum levels of C-reactive protein and lipid profiles in patients with chronic periodontitis and/or coronary heart disease in an ethnic Han population. Quintessence Int (Berlin Germany: 1985).

[22] Nepomuceno R, Pigossi SC, Finoti LS, Orrico SRP, Cirelli JA, Barros SP, Offenbacher S, Scarel-Caminaga RM (2017). Serum lipid levels in patients with periodontal disease: a meta-analysis and meta-regression. J Clin Periodontol.

[23] Sridhar R, Byakod G, Pudakalkatti P, Patil R (2009). A study to evaluate the relationship between periodontitis, cardiovascular disease and serum lipid levels. Int J Dental Hygiene.

[24] Joseph R, Nath SG, Joseraj MG (2011). Elevated plasma homocysteine levels in chronic periodontitis: a hospital-based case-control study. J Periodontol.

[25] Joob B, Wiwanitkit V (2017). Atherogenic index of plasma for the assessment of cardiovascular risk factors. Ann Afr Med.

[26] Xu J, Duan X (2020). Association between periodontitis and hyperlipidaemia: a systematic review and meta-analysis. Clin Exp Pharmacol Physiol.

[27] Gomes-Filho IS, Santos PNP, Cruz SS, Figueiredo A, Trindade SC, Ladeia AM, Cerqueira EMM, Passos-Soares JS, Coelho JMF, Hintz AM (2021). Periodontitis and its higher levels of severity are associated with the triglyceride/high density lipoprotein cholesterol ratio. J Periodontol.

[28] MacDonald CJ, Madika AL, Severi G, Fournier A, Boutron-Ruault MC (2021). Associations between smoking and blood-group, and the risk of dyslipidaemia amongst french women. Sci Rep.

[29] Leite FRM, Nascimento GG, Scheutz F, López R (2018). Effect of smoking on Periodontitis: a systematic review and Meta-regression. Am J Prev Med.

[30] Tonetti MS, Van Dyke TE (2013). Periodontitis and atherosclerotic cardiovascular disease: consensus report of the joint EFP/AAP workshop on Periodontitis and systemic Diseases. J Periodontol.

[31] Griffiths R, Barbour S (2010). Lipoproteins and lipoprotein metabolism in periodontal disease. Clin Lipidol.

[32] Chen S, Lin G, You X, Lei L, Li Y, Lin M, Luo K, Yan F (2014). Hyperlipidemia causes changes in inflammatory responses to periodontal pathogen challenge: implications in acute and chronic infections. Arch Oral Biol.

[33] Nakarai H, Yamashita A, Takagi M, Adachi M, Sugiyama M, Noda H, Katano M, Yamakawa R, Nakayama K, Takumiya H (2011). Periodontal disease and hypertriglyceridemia in japanese subjects: potential association with enhanced lipolysis. Metab Clin Exp.

