# Supplementary material for: Association between atherogenic index of plasma and periodontitis among U.S. adults
Source: BMC Oral Health. 2023 Mar 22;23:166. doi: 10.1186/s12903-023-02853-y (PMC10035221; doi:10.1186/s12903-023-02853-y)
Supplement: Supplementary file 1 — Supplementary Table 1. Periodontitis is classified according to the US CDC-AAP [file 12903_2023_2853_MOESM1_ESM.docx]

Supplementary Table 1. Periodontitis is classified according to the US CDC-AAP

| Group | Definition |
| --- | --- |
| No periodontitis | No evidence of mild, moderate, or severe periodontitis |
| Mild periodontitis | ≥2 interproximal sites with AL ≥3 mm, and ≥2 interproximal sites with PD ≥4 mm (not on same tooth) or one site with PD ≥5 mm |
| Moderate periodontitis | ≥2 interproximal sites with AL ≥4 mm (not on same tooth), or ≥2 interproximal sites with PD ≥5 mm (not on same tooth) |
| Severe periodontitis | ≥2 interproximal sites with AL ≥6 mm (not on same tooth) and ≥1 interproximal site with PD ≥5 mm |

AL: attachment loss; PD: probing depth
