# Supplementary material for: Association between atherogenic index of plasma and periodontitis among U.S. adults
Source: BMC Oral Health. 2023 Mar 22;23:166. doi: 10.1186/s12903-023-02853-y (PMC10035221; doi:10.1186/s12903-023-02853-y)
Supplement: Supplementary file 2 — Supplementary Table 2. Adjusted multinomial logistic regression of AIP with periodontitis [file 12903_2023_2853_MOESM2_ESM.docx]

Supplementary Table 2. Adjusted multinomial logistic regression of AIP with periodontitis

|  | Q1 | Q2 | | Q3 | | Q4 | | *p* for trend |
| --- | --- | --- | --- | --- | --- | --- | --- | --- |
|  |  | OR (95% CI) | *P* | OR (95% CI) | *P* | OR (95% CI) | *P* |  |
| The presence of periodontitis | | | | | | | | |
| Model 1 | Reference | 1.363(1.065,1.744) | 0.015 | 1.598(1.239,2.061) | <0.001 | 1.645(1.262,2.143) | <0.001 | <0.001 |
| Model 2 | Reference | 1.281(0.977,1.680) | 0.072 | 1.414(1.062,1.882) | 0.019 | 1.459(1.097,1.940) | 0.011 | 0.009 |
| Model 3 | Reference | 1.235(0.914,1.669) | 0.161 | 1.160(0.849,1.587) | 0.339 | 1.195(0.883,1.619) | 0.239 | 0.341 |

Model 1: Unadjusted.

Model 2: Adjusted for age and sex.

Model 3: Adjusted for age, sex, BMI, ethnicity, annual family income, education, smoking, and drinking.
