# Supplementary material for: Association between atherogenic index of plasma and periodontitis among U.S. adults
Source: BMC Oral Health. 2023 Mar 22;23:166. doi: 10.1186/s12903-023-02853-y (PMC10035221; doi:10.1186/s12903-023-02853-y)
Supplement: Supplementary file 3 — Supplementary Table 3. Adjusted multinomial logistic regression of AIP with periodontitis in a population aged 40-50 years [file 12903_2023_2853_MOESM3_ESM.docx]

Supplementary Table 3. Adjusted multinomial logistic regression of AIP with periodontitis in a population aged 40-50 years

|  | Q1 | Q2 | | Q3 | | Q4 | | *p* for trend |
| --- | --- | --- | --- | --- | --- | --- | --- | --- |
|  |  | OR (95% CI) | *P* | OR (95% CI) | *P* | OR (95% CI) | *P* |  |
| Model 1 | Reference | 2.149(1.529,3.021) | <0.0001 | 2.834(1.709,4.700) | <0.001 | 3.190(2.005,5.075) | <0.0001 | <0.0001 |
| Model 2 | Reference | 1.903(1.328,2.726) | <0.001 | 2.401(1.377,4.188) | 0.003 | 2.461(1.477,4.098) | <0.001 | 0.005 |
| Model 3 | Reference | 1.532(1.040,2.257) | 0.032 | 1.537(0.879,2.687) | 0.127 | 1.650(0.992,2.744) | 0.053 | 0.162 |

Model 1: Unadjusted.

Model 2: Adjusted for sex.

Model 3: Adjusted for sex, BMI, ethnicity, annual family income, education, smoking, and drinking.
